# Deep Sequencing of Porcine Reproductive and Respiratory Syndrome Virus ORF7: A Promising Tool for Diagnostics and Epidemiologic Surveillance

**DOI:** 10.3390/ani13203223

**Published:** 2023-10-15

**Authors:** Szilvia Jakab, Krisztina Bali, Csongor Freytag, Anna Pataki, Enikő Fehér, Máté Halas, Ákos Jerzsele, István Szabó, Krisztina Szarka, Ádám Bálint, Krisztián Bányai

**Affiliations:** 1 Veterinary Medical Research Institute, Hungária krt. 21., H-1143 Budapest, Hungary; jakab.szilvia@vmri.hun-ren.hu (S.J.); bali.krisztina@vmri.hun-ren.hu (K.B.); anna.pataki@ceva.com (A.P.); feher.eniko@vmri.hun-ren.hu (E.F.); 2 National Laboratory for Infectious Animal Diseases, Antimicrobial Resistance, Veterinary Public Health and Food Chain Safety, Hungária krt. 21., H-1143 Budapest, Hungary; 3 Department of Metagenomics, University of Debrecen, H-4032 Debrecen, Hungary; freytag.csongor@med.unideb.hu (C.F.); szkrisz@med.unideb.hu (K.S.); 4 Prophyl Ltd., H-7700 Mohács, Hungary; mhalas@prophyl.hu; 5 Department of Pharmacology and Toxicology, University of Veterinary Medicine, István u 2, H-1078 Budapest, Hungary; jerzsele.akos@univet.hu; 6 National PRRS Eradication Committee, Keleti Károly u. 24., H-1024 Budapest, Hungary; iszabodr@t-online.hu; 7 Veterinary Diagnostic Directorate, National Food Chain Safety Office, H-1143 Budapest, Hungary; balintad@nebih.gov.hu

**Keywords:** PRRSV-1, PRRSV-2, next-generation sequencing, nucleocapsid, mixed infection, SNV

## Abstract

**Simple Summary:**

Porcine reproductive and respiratory syndrome is a viral disease that causes significant economic losses in many countries worldwide. In this study, we employed a fast, inexpensive, target-specific next-generation sequencing method that can be used for both laboratory diagnosis and epidemiologic monitoring of the disease. We found the protocol to be highly sensitive and potentially effective on clinical serum samples. The method is suitable for the detection of mixed infections and provides a preliminary insight into the microevolution of the causative virus in affected swine herds.

**Abstract:**

Porcine reproductive and respiratory syndrome virus (PRRSV) is a major concern worldwide. Control of PRRSV is a challenging task due to various factors, including the viral diversity and variability. In this study, we evaluated an amplicon library preparation protocol targeting the ORF7 region of both PRRSV species, *Betaarterivirus suid 1* and *Betaarterivirus suid 2*. We designed tailed primers for a two-step PCR procedure that generates ORF7-specific amplicon libraries suitable for use on Illumina sequencers. We tested the method with serum samples containing common laboratory strains and with pooled serum samples (*n* = 15) collected from different pig farms during 2019–2021 in Hungary. Testing spiked serum samples showed that the newly designed method is highly sensitive and detects the viral RNA even at low copy numbers (corresponding to approx. Ct 35). The ORF7 sequences were easily assembled even from clinical samples. Two different sequence variants were identified in five samples, and the Porcilis MLV vaccine strain was identified as the minor variant in four samples. An in-depth analysis of the deep sequencing results revealed numerous polymorphic sites along the ORF7 gene in a total of eight samples, and some sites (positions 12, 165, 219, 225, 315, 345, and 351) were found to be common in several clinical specimens. We conclude that amplicon deep sequencing of a highly conserved region of the PRRSV genome could support both laboratory diagnosis and epidemiologic surveillance of the disease.

## 1. Introduction

Porcine reproductive and respiratory syndrome (PRRS) is a globally spread disease affecting the swine industry. Disease control and prevention is based on vaccination; however, the high genetic diversity of the pathogenic agent, the PRRS virus (PRRSV), poses a constant challenge to the successful implementation of disease elimination programs. First, PRRSVs are separated into two species, *Betaarterivirus suid 1* (PRRSV-1) and *Betaarterivirus suid 2* (PRRSV-2), with only 50–60% sequence similarity between the two species [1]. Second, PRRSV strains within both species are further classified into multiple genetic lineages; all strains are prone to sequence diversification through the accumulation of point mutations and by recombination of homologous genomic fragments [2,3,4,5,6].

Disease control and elimination programs need continuous monitoring of PRRSV status at the herd level, which includes the genetic characterization of circulating viruses [7,8,9,10,11]. Sequencing the ORF5 and ORF7 gene or whole genome analysis has greatly contributed to epidemiologic surveillance since the discovery of PRRSV. In addition, sequencing appears to be very useful when it is required to distinguish between vaccine and field strains in a vaccinated herd or to identify and distinguish resident virus strains in herds from a newly introduced virus [12,13,14,15,16]. From the viewpoint of diagnostics and control measures, it is essential to be prepared for the possibility of multiple PRRSV strains with diverse genetic backgrounds in the herd [17,18,19]. 

Although Sanger sequencing of PCR products has been used traditionally for the genetic characterization of many pathogens, this method may work poorly if genetic variants of the pathogen occur simultaneously in a sample [20,21,22,23,24]. For example, the sequence chromatograms are difficult to evaluate if two sequence variants of PRRSV are present in the sample at similar copy numbers, or a minor variant can be easily overlooked if the copy numbers of co-infecting viruses differ significantly. Although molecular cloning and sequencing multiple plasmid clones may compensate for these disadvantages, this technique is time-consuming and routine diagnostic laboratories are generally not equipped to perform this method. High-throughput sequencing methods that enable massively parallel sequence generation hold the promise that these drawbacks of traditional Sanger sequencing protocols will no longer be relevant [25]. Deep sequencing of target DNA avoids the need for molecular cloning of amplicons and sequencing of multiple plasmid clones. The use of next-generation sequencing (NGS) methods typically yields results in a shorter time and spares diagnostic laboratories from having to perform cumbersome molecular cloning and from building facilities dedicated to the use of genetically modified organisms.

Various NGS approaches have been applied to study PRRSV [26,27,28,29,30,31,32,33,34,35,36,37], and many research groups have recognized that NGS can be very useful for routine testing of PRRSV directly from clinical samples [38,39,40,41,42,43,44]. When evaluating these approaches in published papers, we realized some shortcomings in different methodologies, which included the long sample processing time and the use of expensive reagents such as commercial kits for RNA/DNA concentration and enrichment as well as for library preparation. Moreover, relevant products of some manufacturers are not always available in parts of the world.

In line with these challenges, our objectives were the adaptation and evaluation of a rapid, simple, inexpensive amplicon library preparation protocol specifically for the ORF7 region of PRRSV-1 and PRRSV-2, based on a two-step PCR assay coupled with Illumina short-read sequencing. Archived serum samples and spiked reference strains were tested during the assay development.

## 2. Materials and Methods

### 2.1. Virus Strains and Serum Samples

Prototype strains of PRRSV-1 and PRRSV-2 (Lelystad and VR-2332, respectively) were selected for assay development. The sensitivity of PCR that produced Illumina compatible DNA library was assessed by using spiked samples which were prepared via serial dilutions of the prototype strains in confirmed PRRSV-negative pig serum (dilution range 10^0^ to 10^−9^). All samples were prepared in duplications and then assayed in parallel.

To test the method on clinical samples, we selected 15 archived serum samples that were positive for PRRSV, collected between 2019 and 2021. All strains were previously classified via phylogenetic analysis of the ORF5 sequence and were found to represent ten different clades. All samples in this study were archived as sample pools from various pig farms and processed as such for method development and data analysis.

### 2.2. RNA Isolation and RT-qPCR

Viral RNA was isolated using the NucleoSpin RNA Virus kit (Macherey-Nagel, Düren, Germany) according to the manufacturer’s instruction, and the RNA was stored at −60 °C. To determine the Ct value of spiked and serum samples, RT-qPCR was performed with the virotype PRRSV RT-PCR Kit (Qiagen, Hilden, Germany) according to the instructions of the manufacturer. 

### 2.3. Design of ORF7 Primers

**Figure 1:**
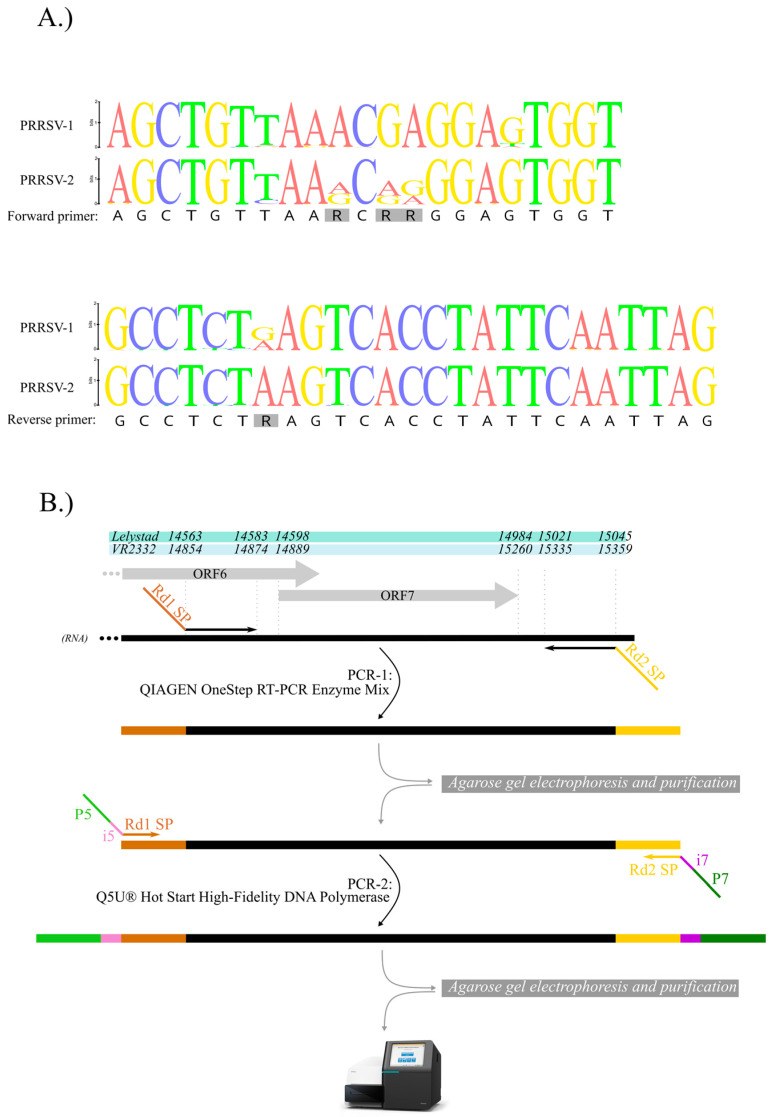
(**A**) Nucleotide sequence alignment of PRRSV-2 (*n* = 466) and PRRSV-1 (*n* = 90) complete genome sequences showing the newly designed ORF7-specific amplicon sequencing primer regions. In the sequence logo, the relative size of characters indicates the frequency of the characters (i.e., nucleotides) in the alignment. (**B**) Position of the amplified ORF7 product on both reference genomes (Lelystad and VR-2332), respectively, and the schematic presentation of the two-step RT-PCR-based library preparation protocol.

To design a broad-range ORF7 primer pair, we compiled a whole-genome sequence dataset comprising 466 PRRSV-2 and 90 PRRSV-1 GenBank records. The idea to obtain full-length ORF7 genes led us to search for a conserved region located outside the target gene in both PRRSV-1 and PRRSV-2 sequences (Figure 1). The final ORF7 product is amplified from position 14,563–15,046 and 14,854–15,360 relative to Lelystad (accession number, M96262) and VR-2332 (accession number, U87392), respectively.

### 2.4. Two-Step RT-PCR-Based Amplicon Library Preparation

PCR-1 was designed to amplify the target genomic region from the RNA extracts of clinical samples and to add the universal Illumina sequencing primer. PCR-2 was designed to add Illumina flow-cell hybridization signals and unique sample identifier (i.e., index) sequences selected from a recommended index set of Illumina. 

PCR-1 consisted of a one-step RT-PCR (using QIAGEN OneStep RT-PCR Kit, Qiagen, Hilden, Germany), employing degenerate forward and reverse primers (forward, 5′–*TCGTCGGCAGCGTCAGATGTGTATAAGAGACAG*gtcaa**AGCTGTTAARCRRGGAGTGGT**–3′; reverse, 5′–*GTCTCGTGGGCTCGGAGATGTGTATAAGAGACAG*catga**CTAATTGAATAGGTGACTYAGAGGC**–3′ (italic—Illumina-compatible sequencing primer binding sites Rd1 SP and Rd2 SP; bold—PRRSV-1 and PRRSV-2 ORF7-region-specific section; Figure 1). The reaction was performed according to the manufacturer’s instruction in a final volume of 25 µL. The reaction mixture comprised 1 µL enzyme mix, 4 U RiboLock RNase Inhibitor (Thermo Scientific™, Waltham, MA, USA), the primer pair at 0.6 µM concentrations, and 2 µL template RNA. The PCR cycling condition started at 50 °C for 30 min and 95 °C for 15 min, followed by 35 cycles of 94 °C for 30 s, 57 °C for 30 s, and 72 °C for 1 min; the final extension lasted for 5 min at 72 °C.

PCR-2 was performed to add Illumina flow cell adapters (P5 and P7) and index sequences (Nextera XT Index Kit v2 Set C) to the PCR-1 products (Figure 1). In this round, the forward and reverse indexing primers, 5′–AATGATACGGCGACCACCGAGATCTACACxxxx*TCGTCGGCAGCGTC*–3′ (P5; xxxx: i5 index; italic—part of sequencing primer binding sites) and 5′–CAAGCAGAAGACGGCATACGAGATzzzz*GTCTCGTGGGCTCGG*–3′ (P7; zzzz: i7 index; italic—part of sequencing primer binding sites), were used for the amplification. To minimize the chemistry-associated mutations, PCR-2 was executed with Q5U^®^ Hot Start High-Fidelity DNA Polymerase (NEB, Frankfurt am Main, Germany) according to the manufacturer’s instructions in a final volume of 25 µL with final primer concentration of 0.5 µM and using 5 µL undiluted gel-purified product of PCR-1 as template. PCR cycling condition was initiated at 98 °C at 30 s, followed by 30 cycles of 98 °C for 20 s, 55 °C for 15 s, and 72 °C for 1 min; the final extension lasted for 5 min at 72 °C.

After PCR-1 and PCR-2, the amplicons were analyzed on 1% and 1.5% agarose gel, respectively. The bands in the expected size (~550 bp for PCR-1 and ~640 bp for PCR-2) were excised and purified from the gel using the FavorPrep GEL/PCR Purfication Mini Kit (Favorgen, Ping Tung, Taiwan) to eliminate primer dimers and residual components of the reaction mixtures.

The purified DNA from PCR-2 was measured and prepared to run on an Illumina sequencer. We used an iSeq 100 Sequencing System with iSeq 100 i1 Reagent v2 (300-cycle) kit for initial analyses and an Illumina^®^ MiSeq platform with MiSeq Reagent Kit v3 (600-cycle) using paired-end procedure to obtain the full-length ORF7 sequences from clinical samples. Although iSeq was unable to produce the full-length amplicon, it served as a good proxy to assess the assay sensitivity and helped adequately parametrize downstream processes.

### 2.5. Analysis of NGS Data

All NGS data analyses were conducted in Geneious Prime^®^ (version 2022.2.2). Prior to read assembly, short reads (<75 bp) were discarded, and ORF7-specific primers and low-quality bases (minimum Phred score of Q30) were removed at both ends using the BBDuk plugin in Geneious. In case of spiked samples, the consensus sequences were created by using GenBank reference sequences of Lelystad and VR-2332, respectively. To characterize the consensus sequences of PRRSV variants in mixed clinical samples, we performed parallel mapping to the first constructed consensus and to the Porcilis MLV-DV strain (accession number, KJ127878), as inferred from the dissimilar reads. To estimate the proportion of PRRSV variants in mixed serum samples, we identified the nucleotide positions that differed between the sequence pairs, and afterwards, we calculated the mean SNV variant frequencies for these sites from the contig file of the major consensus. To determine the intra-strain variation of the serum samples, we conducted an SNV search, where variant calling was performed with minimum variant frequency of 10%.

Newly generated ORF7 sequences were deposited in GenBank. Accession numbers are as follows: OR427017-OR427035.

## 3. Results

### 3.1. Assay Specificity

Primers were designed based on the ORF7 gene. A total of 556 genome sequences were aligned to find potential target regions. These regions were located upstream and downstream of the ORF7 so that the designed primers seemed to be suitable for the amplification of the full-length ORF7 gene (Figure 1). Following the in silico analysis, we tested the specificity, the sensitivity, and the reactivity spectrum of the primers in a two-step PCR assay.

**Figure 2:**
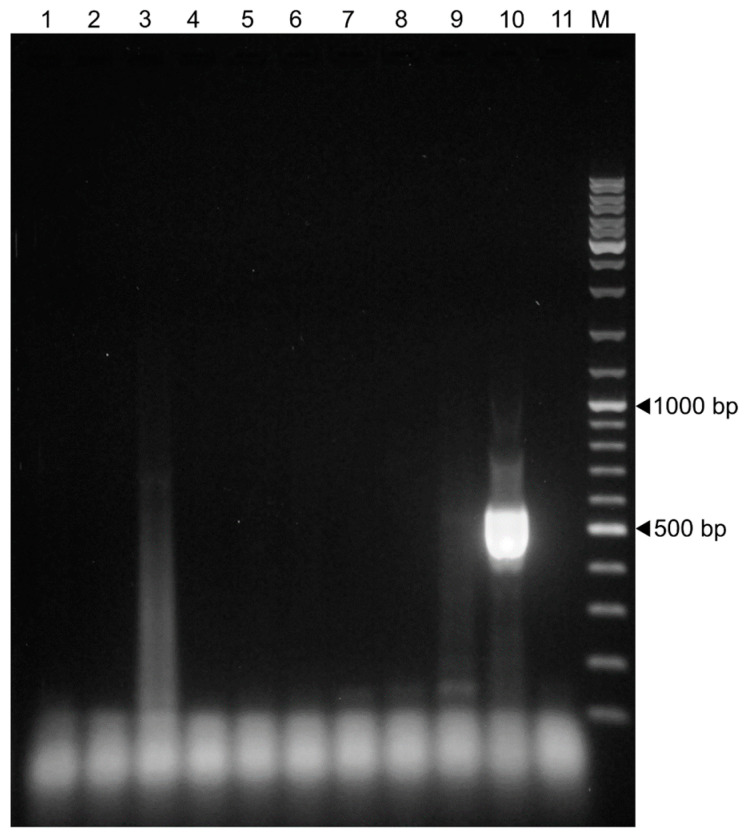
Result of the ORF7 amplicon deep sequencing PCR assay conducted with various swine viruses. Lanes 1–9 refer to suid herpesvirus 1 (lane 1), porcine parvovirus 1 (lane 2), porcine epidemic diarrhea virus (lane 3), classical swine fever virus (lane 4), porcine teschovirus (lane 5), transmissible gastroenteritis virus (lane 6), porcine circovirus 2 (lane 7), atypical porcine pestivirus (lane 8), and porcine rotavirus (lane 9); lanes 10 and 11 refer to positive and non-template control, respectively; M: GeneRuler DNA Ladder Mix.

The assay was evaluated by using two reference strains, Lelystad (PRRSV-1) and VR-2332 (PRRSV-2). Although sequencing on iSeq100 equipment did not generate the full-length ORF7 genes, this approach permitted the verification of the assay design, clearly demonstrating that the incorporated index sequences were accurate; we verified the specificity and sensitivity data of our newly developed PCR assay, showing that the expected consensus sequences were generated. In this phase, additional swine viruses were tested in the PCR protocol using extracted nucleic acids from laboratory isolates of suid herpesvirus 1, porcine parvovirus 1, porcine epidemic diarrhea virus, classical swine fever virus, porcine teschovirus, transmissible gastroenteritis virus, porcine circovirus 2, atypical porcine pestivirus, and porcine rotavirus. Only nucleic acids extracted from the PRRSV isolate provided PCR products of the expected size. Non-specific amplicons other than primer dimers were not observed for the selected viruses in agarose gel, except for a smear that appeared in a single test virus, porcine epidemic diarrhea virus (Figure 2).

### 3.2. Assay Sensitivity

The sensitivity was tested through serial dilutions of serum specimens spiked with the two laboratory strains. The Ct values obtained using a commonly used PRRSV real-time RT-PCR kit were used to express the quantity of viral RNA. In this experiment, we used a range of dilutions between 10^0^ and 10^−9^. The Ct values for undiluted samples were 13.2 (Lelystad) and 12.7 (VR-2332). PCR products at the end of the second PCR round were seen in agarose gel, even in samples diluted to 10^−6^; this dilution of viral RNA corresponded to Ct 34.7 for Lelystad and Ct 34.4 for VR-2332. As expected, the assembled sequences were consistent with the consensus sequences of Lelystad and VR-2332, respectively.

**Figure 3:**
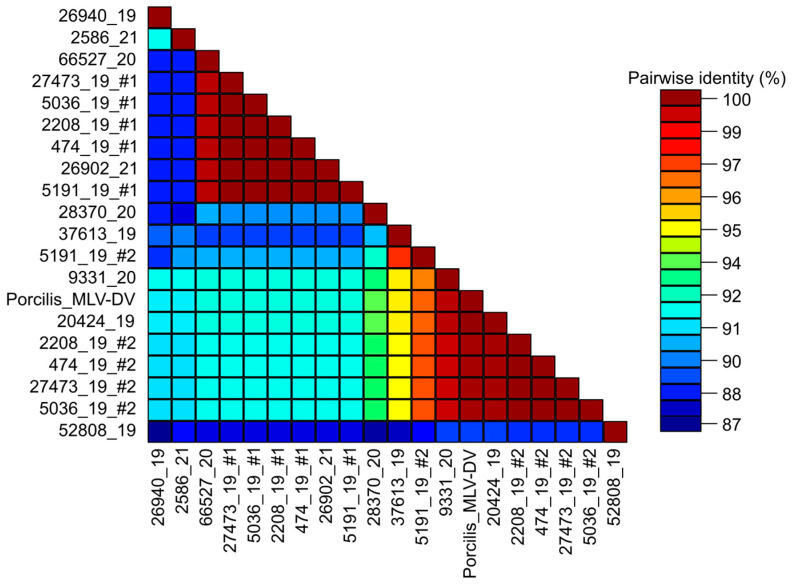
Pairwise nucleotide identities based on the ORF7 gene sequences of the test serum samples and the Porcilis MLV-DV strain. Some samples contained two variants. The co-infecting major and minor variants are marked with #1 and #2, respectively.

After the specificity and sensitivity with selected reference strains were found to be satisfactory, the sensitivity testing was extended to clinical specimens, providing the opportunity to test reactivity with different PRRSV clades as well. Strain selection from the archived serum samples was carried out based on previously identified ORF5 sequences. Some of the selected samples represented the same ORF5 lineages (Table 1). The Ct values ranged from 16.5 to 31.6, although the sample with the highest Ct value did not produce specific amplicons and was not further analyzed. Another sample with a similar Ct value (29.1) and all other samples yielded amplicons of the expected size following the second-round PCR and were subjected to sequencing. The obtained ORF7 sequences could be classified into eight phylogenetic clades. The nucleotide similarity of the ORF7 among selected strains ranged from 87.4% to 100% (Figure 3).

### 3.3. Analysis of Sequence Diversity

Deep sequencing uncovered the population structure of the amplified viral RNA sequences.

When selecting the serum samples, we expected that a single strain was present in all the selected samples; however, additional PRRSV sequences were assembled in a subset of samples, suggesting mixed infection with different strains in those specimens. The analysis of the deep sequencing data identified five pooled samples (474_19, 5036_19, 2208_19, 5191_19, and 27473_19) in which multiple PRRSV strains were present simultaneously. The estimated proportion of the identified strains in these five sample pools showed some variations (Table 1). The pairwise nucleotide identities between members of the sequence pairs in any samples fell below 93%, and in each case, the obtained sequences of the pairs could be clustered into different phylogenetic clades. In four samples, the co-infecting minor virus strain was the vaccine origin Porcilis MLV-DV, which shared >99% nucleotide identity values in pairwise comparisons with the sequences assembled from the serum pools. In the fifth mixed sample, the co-infecting minor virus variant shared only 97.3% nucleotide identity with the Porcilis vaccine strain.

**Figure 4:**
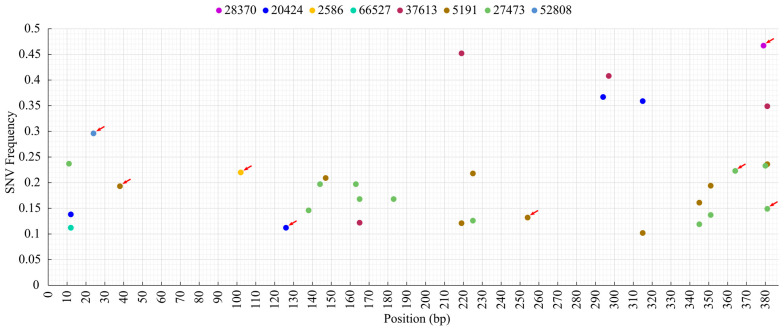
Frequency and distribution of SNV sites within the ORF7. SNVs leading to amino acid changes are marked with red arrows.

Due to the relatively high sequencing depth of amplicons (average, 26,402×; minimum, 202×; maximum, 149,865×), the single nucleotide variation (SNV) could be evaluated in all samples. We identified SNVs in eight samples. The number of polymorphic sites was 33; of these, seven sites (position 12, 165, 219, 225, 315, 345, 351) were shared in at least two independent samples, and one (position 381) was shared in three samples. The proportion of minority SNVs causing changes in the amino acid sequence of the nucleocapsid was 27.3% (Figure 3). All these non-synonymous substitutions were located in unique positions along the ORF7. The highest numbers of SNVs were detected in samples 5191_19 (*n* = 10) and 27473_19 (*n* = 12), representing the Reprocyc and the Spanish clades, respectively. The frequency of polymorphic sites ranged from 0.3 to 3.1%, and the proportion of the minor sequence variants ranged between 10.2% and 46.7% (Figure 4).

## 4. Discussion

Next-generation sequencing has revolutionized life sciences. In parallel with the significant reduction of ‘per base pair’ sequencing costs, veterinary diagnostics has also benefited from the methodological developments and some areas, such as pathogen identification and characterization, have become major areas for the routine use of NGS [45,46,47,48,49,50]. Amplicon sequencing using NGS platforms serves as a fast, sensitive, and inexpensive way to characterize genomic regions of interest or conduct whole-genome sequencing via an ARTIC-type tiling amplicon scheme [51,52]. PCR-based library preparation approaches that include target-specific primer sequences with incorporated adapter tails lowered the handling time and the cost of library generation and reduced the required DNA quantity for successful sequencing via various NGS chemistries [53,54,55,56]. Although producing amplicon libraries exclusively through PCR is a well-established method, the utilization of this approach in veterinary science is largely restricted to profiling microbial communities [20,57,58,59,60,61].

An ORF7-gene-based amplicon sequencing assay was evaluated in this study. We designed a two-step PCR protocol that permitted the amplification of the target region in the first round and incorporated additional Illumina-specific sequences in the second round. Importantly, the target-specific primers were designed to amplify both PRRSV-1 and PRRSV-2 strains, and this was confirmed in the initial experiments using selected reference strains. Moreover, we verified the assay specificity by testing viral strains causing major viral diseases in swine. As expected, no specific bands were amplified in the PCR-1 reaction. The functionality of PCR-2 was evaluated in an Illumina sequencer (iSeq100) that showed that Illumina-specific sequences were incorporated during the two rounds of PCR. Then, we tested the sensitivity of the two-step library preparation protocol. We chose to use Ct values as the primary endpoint for sensitivity, given that it is a major laboratory indicator of viral load in the molecular diagnosis of PRRSV infection. The Ct values of spiked samples of Lelystad and VR-2332 isolates ranged from 13.2 to 34.7 and 12.7 to 34.4, respectively. Our results showed that amplicons of the expected size were generated, and full-length ORF7 sequences could be assembled even from low-copy-number RNA samples of reference PRRSV-1 and PRRSV-2 strains. 

Although whole-genome sequencing of viruses has become a routine task in many veterinary diagnostic and epidemiologic laboratories worldwide, the approach has some drawbacks compared with partial-gene-based NGS techniques, and it is advisable to adapt different approaches depending on the actual needs. Thus, there is no doubt that whole-genome sequencing of viruses is more suitable for genetic characterization of circulating strains and is the preferred method for the identification of sequence variants, recombinants, or putative vaccine-derived strains [49,62]. However, the high degree of sequence divergence of many viruses may prevent the development and application of universal, broad-spectrum amplification methods suitable for generating complete viral genomes from tissue samples or virus cultures; these are obstacles that need to be taken into account when considering the use of NGS in whole-genome sequencing. Additionally, the viral genome copy number is often too low, which may prevent the use of whole-genome sequencing protocols for routine testing of clinical specimens. We believe that deep sequencing of amplicons generated from parts of the genome may be a good proxy in order to obtain initial data concerning the infecting virus species and genotypes. In this respect, PRRSV is a good candidate, because ongoing disease eradication programs require continuous monitoring of circulating field- and vaccine strains. Additionally, the ORF7 genomic region of PRRSV is a common target for diagnostic tests and, with some negligible limitations, can be utilized in epidemiological investigations [4,13,63,64,65,66,67]. The assay format presented here is generally more sensitive and is considerably faster and less expensive than the whole-genome sequencing protocols used in virus detection and identification. It has been demonstrated that whole-genome sequencing may fail when PRRSV-positive serum samples above Ct 25 to 30 are tested. In contrast, our assay was sensitive enough to amplify the target gene from samples with a Ct value of 35 (or so), which is close to the sensitivity of real-time PCR assays that typically target very short gene fragments, and at least two orders of magnitude more sensitive than published deep sequencing protocols designed to study whole genomes of PRRSV [26,27,38,39,40]. Moreover, the costs and hands-on time needed to generate library DNA in our protocol is essentially the same as the expenses and time required to perform a nested RT-PCR assay.

To further evaluate our method, ORF7 amplicon sequencing was used to test serum samples containing diverse PRRSV strains that had circulated in Hungary between 2019 and 2021. The Ct values recorded for these serum samples varied between 16.5 and 31.6, and all but one sample (30156_20) yielded full-length ORF7 sequences. In the case of 30156_20, the Ct value was close to the detection limit, and it is possible that the strain in this sample represented a genetic lineage that may have carried some mutations in key positions of the primer binding region that prevented the amplification, or, more likely the RNA that was present in low copy numbers could have degraded over time during sample storage and repeated freezing and thawing cycles.

Subsequent analysis of the deep sequencing data identified five pooled samples in which multiple PRRSV variants were simultaneously identified. In four samples, the co-infecting minor PRRSV strain was the vaccine-origin Porcilis MLV-DV, while in the fifth sample, the co-infecting minor virus variant shared 97.3% identity with the Porcilis vaccine strain. This similarity of the minor variants in two samples (2208_19 and 474_19) was not unexpected given that the farms where these specimens were collected used the Porcilis vaccine during the process of the PRRS elimination program. In fact, this co-occurrence of vaccine and wild type strains in herds where PRRSV control measures were taken has been reported in previous studies [17,19,26,40].

In addition to the identification of vaccine and field strain co-infections, deep sequencing delivered insights into the intra-strain variation in PRRSV positive samples. Although the implications of this snapshot on the intra-strain variability were limited because serum samples were pooled, the findings convincingly demonstrated another aspect of the advantages of amplicon deep sequencing. Our analyses showed that eight serum samples harbored polymorphic sites unevenly distributed along the ORF7 region. In other studies that described full-length PRRSV genomes, ORF7 was among the genomic regions where the fewest SNVs were observed [28,33,68]. This discrepancy with our findings suggests that the observed variation in the SNV frequencies in a relatively conserved genomic region may arise from sequence divergence between pigs, rather than from variation within the same pig. However, given that samples were pooled in our study, it is impossible to confirm this hypothesis. Nonetheless, all these results provide preliminary findings about the microevolution of the ORF7 at the herd level and justify the need to initiate additional targeted deep sequencing projects in future elimination programs. Based on the distribution and frequency of SNVs, it appears that different PRRSV strains may be affected by different evolutionary drivers, but more representative sampling is needed to better describe and understand these processes.

## 5. Conclusions

In this study, we presented the preliminary evaluation of a novel ORF7-gene-based amplicon deep sequencing method not tested so far for PRRSV. Although the number of samples available to represent the local diversity of PRRSV strains was limited at the time of the development of the assay due to the long-standing and successful national PRRS eradication program in Hungary, the ORF7 amplicon deep sequencing method had some advantages over traditional sequencing technologies. In brief, our method (i) is able to detect both PRRSV-1 and PRRSV-2, (ii) is a fast, inexpensive, sensitive, and targeted tool for detecting very low copy numbers of viral RNA from clinical samples, (iii) shows broad reactivity with recently circulating lineages, (iv) detects multiple strains co-circulating in any swine herds and, in most instances, is suitable for discriminating field- and vaccine strains in ongoing disease eradication projects, and (v) uncovers the (micro)evolutionary processes of the target gene. We believe that the method can be readily adapted by routine diagnostic laboratories and will be very useful during PRRS elimination programs, particularly in the phase of elimination, when wild type and vaccine strain co-infections frequently occur and their co-circulation needs to be monitored in vaccinated herds.

## Figures and Tables

**Table 1:** General data of the serum samples collected from multiple Hungarian pig farms and a summary of the ORF7 amplicon sequencing results.

Sample	Ct Value	ORF5 Clade	Filtered Reads	Sequencing Depth (X)	Average SNV (%)	
					Major Variant	Minor Variant *
26940_19	20.9	1A	502,722	149,864	-	-
2208_19	27.8	1E	22,306	1029	75	25
28370_20	26.5	1E	109,948	41,137	-	-
20424_19	21.5	1F	154,252	38,505	-	-
26902_21	19.1	1G	93,512	38,471	-	-
2586_21	16.5	1G	33,762	4351	-	-
474_19	22.3	1G	15,406	305	78	22
66527_20	22.0	Porcilis	172,782	67,857	-	-
9331_20	24.4	Porcilis	105,896	13,271	-	-
37613_19	22.6	Porcilis-like	31,070	6245	-	-
5191_19	27.8	Reprocyc	35,600	5846	53	47
27473_19	29.1	Spanish	5330	1605	58	42
5036_19 °	25.8	Spanish	898	202	62	38
30156_20	31.6	3D	-	-	-	-
52808_19	26.5	3F	6856	939	-	-

° Reads were filtered with minimum Phred score of Q20. * Porcilis MLV was identified as minor variant in four samples and a Porcilis MLV-related variant was identified in the fifth specimen (5191_19).

## Data Availability

Sequence data were deposited in GenBank (OR427017-OR427035).
